# Developing Pediatric medication education for underserved families: A patient-centric product development approach focusing on medication instructions, side effects, and administration techniques

**DOI:** 10.1016/j.pecinn.2026.100507

**Published:** 2026-09-28

**Authors:** Tiranun Rungvivatjarus, Nayely Cazares, Letzy Vargas, Elizabeth Haddad, Michael Henne, Gale Romanowski, Kyung E. Rhee

**Affiliations:** a Department of Pediatrics, University of California San Diego, 9461 Gilman Dr, La Jolla, CA 92123, USA; b Rady Children's Hospital, 3020 Children's Way, San Diego, CA 92123, USA; c Columbia University Mailman School of Public Health, 722 W 168^th^ St, New York, NY 10032, USA; d Skaggs School of Pharmacy and Pharmaceutical Sciences, University of California San Diego, 9255 Pharmancy Ln, La Jolla, CA 92093, USA; e University of California San Diego, 9500 Gilman Dr, La Jolla, CA 92093, USA

**Keywords:** Health education, Health literacy, Medication education, Medication safety, Community research

## Abstract

**Objective:**

To utilize a patient-centric product development (PCPD) approach to create three medication education videos for families in underserved communities.

**Methods:**

PCPD was used to create the educational videos, involving prototype video development and iterative parent feedback for video refinement. Video topics include: (1) medication instructions, (2) side effects, and (3) administration techniques. Parents were recruited from underserved communities in San Diego.

**Results:**

We collected fifty surveys from English-speaking parents and fifty surveys from Spanish-speaking parents. Median post-video knowledge scores statistically increased from pre-video scores across all three videos (*p* ≤ 0.001). Parents reported that video content was easy to understand, increased their confidence in medication administration, taught them a new useful fact or skill, and that they would recommend these videos to other parents.

**Conclusion:**

We successfully created pediatric medication education resources that parents comprehend, find useful, and are satisfied with for managing their children's medications. Future steps include expanding the availability of our videos in multiple languages and evaluating their impact on medication delivery and health outcomes.

**Innovation:**

This study advances the field by adapting the PCPD framework – traditionally used in drug and medical device development – to co-create patient-facing educational tools, shifting from a provider-driven model to one that is patient/consumer-centric. By identifying unmet informational needs and translating them into a structured, parent-informed audio-visual format, this study contributes both novel content and a transferable methodological approach for future community health education initiatives.

## Highlights

• PCPD framework was adapted to co-create pediatric medication education videos.
• Multidisciplinary teams and parent councils ensured cultural and linguistic relevance.
• Parents find our videos comprehensible and useful in their medication management.
• Our methodology shifted from provider-driven to a parent-centric education creation.

## 1 Introduction

Underserved populations refer to communities that include members of minority populations or individuals who have experienced health disparities. [1] A growing body of research suggests that health literacy may be a key factor contributing to these health disparities. [2], [3] Although low health literacy affects people of all socio-demographic groups, it disproportionally affects underserved populations. [4] Inadequate health literacy - defined as the inability to read and understand health-related material and instructions - has been shown to adversely affect medication administration, medication adherence, and health management. [5], [6], [7] These challenges contribute to increased healthcare utilization and cost the United States healthcare system an estimated $238 billion annually. [8]

Existing educational strategies, including printed materials, are not always successful in providing medication information that is understandable, accessible, and actionable for families. [9], [10] The current guidelines for patient education materials state that they should be written at a fifth- or sixth- grade reading level. [11] However, many written educational materials demand literacy levels that exceed these recommendations. [12], [13], [14] Furthermore, comprehension (i.e., the ability to read and understand written health-related information sufficiently to make informed decisions) is a core component of health literacy that is often measured in a very limited scope. [15] In our previous focus group study, we found that families managing pediatric medications in underserved communities faced common barriers to comprehension, including the use of medical jargon, lack of accessible pediatric-specific medication information, and vague medication instructions. Common instructions, such as “three times a day,” were not as clear to parents as providers believed. Ultimately, parents wanted simplified terminology and digestible information, including medication side effects, dosing frequency, and administration techniques for bad tasting medications. [16] These findings suggest that currently available educational resources do not consistently address the practical information needs of caregivers and may leave important medication knowledge gaps unaddressed.

In addition, many existing pediatric medication education material focus on specific diseases or treatment programs, limiting their applicability across diverse patient populations. [17], [18], [19], [20], [21] However, fundamental medication management challenges are often shared regardless of a child's diagnosis. Studies have documented that pediatric medication errors often occur due to deficiencies in fundamental medication management skills, including accurate dosing of liquid medication, adhering to appropriate dosing intervals, interpreting instructions, and premature discontinuation of the drug. [22], [23], [24], [25], [26], [27], [28], [29], [30] Additionally, medications most implicated in pediatric medication errors at home are analgesics and over-the-counter cough and cold medications, rather than disease-specific medications. [25], [31], [32] Developing accessible, comprehensible, and broadly applicable medication education tailored to underserved communities is thus critical to improving pediatric health outcomes.

In this study, using a patient-centric product development (PCPD) methodology, we aimed to create three medication education videos for parents living in underserved communities. The video topics included medication instruction, side effects, and administration. We hypothesized that parents' comprehension of these topics would increase after watching the videos, and that satisfaction and value would be high. By enhancing access to clear, concise information, we hope to reduce health literacy gaps, thereby decreasing instances of suboptimal health management, medication errors, and non-compliance.

## 2 Methods

### 2.1 Educational videos development

To develop educational materials that can improve parent management of medications, we used a patient-centric product development (PCPD) methodology, which is a customer-focused product development process. PCPD uses a systematic approach to incorporate patient perspectives and preferences into the design and production of a product. [33] Each stage of the product development lifecycle often has a patient-focused design consideration with opportunities for direct patient engagement and iterative product improvement. Patient-centricity can encompass specific patient engagement-based activities that involve patients sharing their experiences, needs, and priorities through qualitative and quantitative research methods such as interviews, focus groups, and surveys. [33] This methodology has been mainly employed by biopharmaceutical companies in drug design since patients are an important stakeholder in achieving desired therapeutic outcomes. [34] Outside of drug development, this concept has been utilized in medical device and physician development curriculum design. [35], [36]

We utilized PCPD methodology to create desired educational materials that best serve our parent population. Previous studies have shown video-based education improves knowledge for low health literacy populations compared to standard methods, but often lack iterative patient input. [17], [18], [37], [38], [39], [40], [41], [42] Our video topics were selected based on parents' reported knowledge gaps from a prior focus group study: (1) understanding medication instructions, (2) side effects of commonly prescribed and over-the-counter pediatric medications, and (3) medication administration techniques, which have not been previously evaluated in video format. [16] The video content and scripts were developed through a collaborative workgroup consisting of two pharmacists, one child life specialist, one nurse, our local family advisory council, two pediatricians, and two health literacy experts. To develop each video, a set of learning objectives and topics were established based on common medication management challenges identified in literature, clinical practice, and content expert experience (Table 1). These objectives guided the creation of detailed scripts, which included plain-language explanations, visuals, and story-based clinical vignettes. Video scripts were then reviewed by the Family Advisory Council (FAC) for feedback to improve clarity, relevance, and comprehension. Founded in 2010, the FAC is a long-standing institutional body comprising approximately twenty-five members, including hospital leadership (e.g. Chief Nursing Officer, and Safety Officer), hospital employees, and parents of children treated at the facility. During a dedicated session, the research team presented the proposed video topics and scripts; members who could not attend provided feedback via email to ensure a comprehensive review from the entire council.

**Table 1:** Educational video topics and key learning objectives.

Video Topic	Key Learning Objectives
Medication Instructions	• Understanding medication frequency and the timing of doses (e.g., “three times daily”). • Understanding medication storage instructions (refrigeration versus room temperature). • Understanding when to take medications with food versus empty stomach.
Side Effects	• Identifying common and serious side effects of the following medications: o Amoxicillin o Cephalexin o Albuterol o Acetaminophen o Ibuprofen o Polyethylene Glycol o Ondansetron o Cetirizine o Loratadine
Administration	• Identifying techniques to help children of various ages take medications o Using distraction or consistent routine with infants and toddlers o Providing choices for school-age children o Discussing health consequences of medication noncompliance with teens • Naming general tips to encourage medication compliance based on various forms of medication (e.g., pills, tablets, liquid)

Following this collaborative review and feedback sessions, the research team created three prototype educational videos in both English and Spanish using animation video creation software (Vyond™, © 2024 GoAnimate, Inc.). To keep the videos concise, we included nine commonly prescribed and over-the-counter medications within a single side effects video in each language (English and Spanish). Prescription frequency data from our children's hospital–affiliated outpatient pharmacy was used to identify the most commonly prescribed medications among children aged 0–18 years.

Focus group participants were recruited from an underserved community of San Diego, via recruitment events at our four community partner sites (Copley-Price YMCA, Children's Primary Care Medical Group El Cajon, Metro Villas Complex, and Rady Children's Hospital Outpatient Clinic). Study eligibility requirements were determined by a screening conducted by a research team member. Parents were selected to participate if they were: 1) the family member or caregiver of a child 0–12 years of age who had taken or was taking prescribed medications at the time of recruitment, 2) the primary person who managed or co-managed the child(ren)’s medication(s), and 3) identified as having a primary language of English or Spanish. A unique identification number (study ID) was used to maintain confidentiality. Eligible participants were invited by the research team to participate in several 60-to-90-min focus group discussions that were conducted in either English or Spanish. Ten English-speaking and nine Spanish-speaking parents were recruited for the focus group portion of the study. We conducted two iterative focus groups sessions in each language to revise and improve the videos. Each focus group was facilitated by two research team members, with one taking notes on parents' feedback and the other navigating the videos and moderating feedback discussions.

At the beginning of each initial focus group, participants completed a demographic survey. We presented three educational videos, after which parents provided feedback to enhance their quality and guide further improvements. English videos were reviewed by English-speaking parents, while Spanish videos were reviewed by Spanish-speaking families, enabling the latter to provide specific feedback to the Spanish videos that ensured linguistic accuracy and cultural appropriateness. Feedback from English- and Spanish-speaking parents was collected and reviewed by the multidisciplinary research team to ensure content accuracy and to determine the number and scope of revisions before incorporation into the videos. A second round of focus groups was conducted with the same group of parents to review the revised videos. This approach aimed to maintain consistency in comments and feedback throughout the editing process. Additional feedback from the second round of focus groups was collected and incorporated to refine the final version of the educational videos. The final version of the medication instruction and side effects video were 6 min in length, while the medication administration video was 6 min and 44 s (Fig. 1, Fig. 2, Fig. 3). All participants received a $50 gift card after each round of the focus group.

**Fig. 1:**
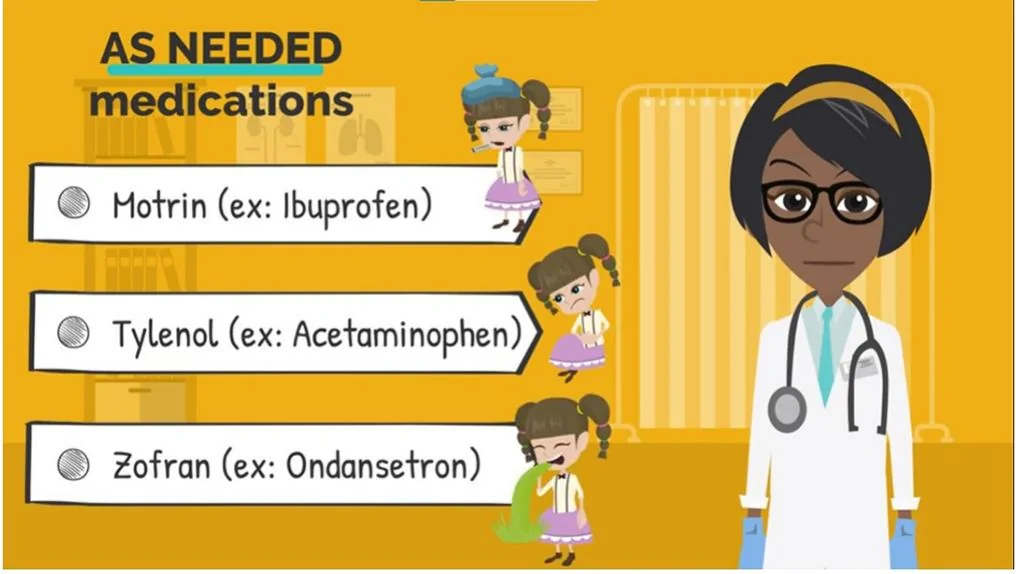
Still image of Medication Instruction video. Video can be accessed electronically via this link (English: https://vimeo.com/1221913105/cce7c88b50?share=copy Spanish: https://vimeo.com/1221913106/dfb753ab30?share=copy)

**Fig. 2:**
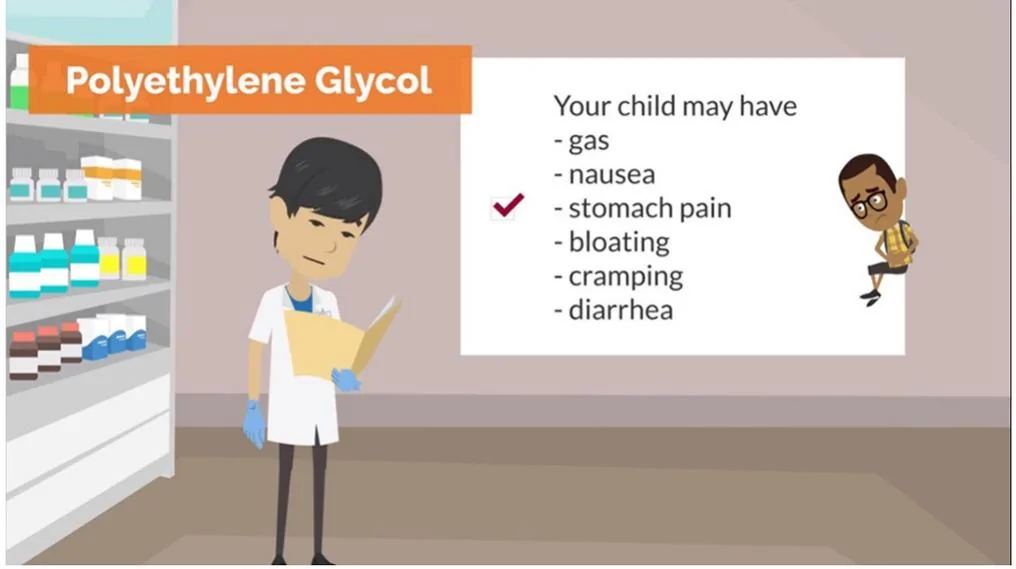
Still image of Medication Side Effects video. Video can be accessed electronically via this link (English: https://vimeo.com/1221913083/8c55568a7a?share=copy Spanish: https://vimeo.com/1221913084/3910767fbc?share=copy&fl=cl&fe=ci)

**Fig. 3:**
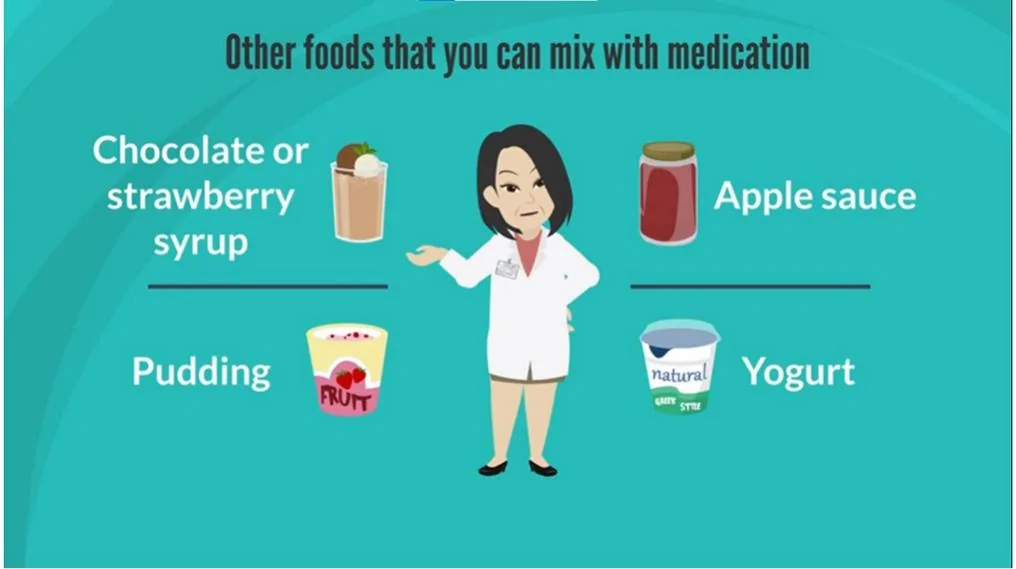
Still image of Medication Administration video. Video can be accessed electronically via this link (English:  https://vimeo.com/1221913081/7483d4af49?share=copy Spanish: https://vimeo.com/1221913082/01bd7d48db?share=copy)

### 2.2 Educational videos evaluation

To evaluate the utility, content, and comprehensibility of these medication videos, 100 parents (50 English-speaking and 50 Spanish-speaking parents) were recruited. Parents were recruited in-person from Rady Children's Hospital and our four community partner sites. Parents who participated in the development of the videos were ineligible for this portion of the study. Parents were eligible to participate if they were: 1) the family member or caregiver of a child 0–12 years of age who had taken or was taking prescribed medications at the time of recruitment, 2) the primary person who managed or co-managed the child(ren)’s medication(s), and 3) identified as having a primary language of English or Spanish. Upon meeting eligibility criteria and providing informed consent for participation in the study, parents completed a baseline survey regarding medication instruction knowledge, medication side effects knowledge, medication administration knowledge, and demographic information (Supplemental). The knowledge questions were designed to align with the learning objectives of the medication education videos and reviewed by pediatric healthcare experts (pharmacist, child life specialist, general pediatrician, and hospital medicine pediatrician) for face and content validity. We ensured clarity, relevance, and coverage of essential knowledge through expert feedback and pilot testing with a small group of parents. After completion of the pre-survey study, parents were presented with the three educational videos (medication instruction, side effects, and administration) which were accessible through a REDCap (Research Electronic Data Capture) survey form. [43], [44] Survey and video links were made available via a QR code that could be accessed through participant's mobile phones or the research team's tablets, providing flexibility and convenience. Upon completion of viewing each video, another follow-up survey was provided to assess changes in medication instruction knowledge, medication side effects knowledge, and medication administration knowledge. A follow-up survey included questions on content comprehension, confidence in medication administration, usefulness (e.g., learned skills or knowledge), and satisfaction (e.g., likelihood of recommending to other parents). Each video knowledge assessment contains 4–5 multiple-choice questions. Participants were given 1 point for each question they answered correctly to create a summary score. The same questions were asked before and after the video viewing. Participants were not provided with an answer key or summary score. All participants received a $30 gift card for completing the survey. This study was approved by the University of California San Diego Institutional Review Board.

### 2.3 Measures

We assessed parents' knowledge gained from each of the educational videos through multiple-choice questions, a commonly used knowledge assessment tool in pediatrics. [45], [46], [47] The Medication Instruction video had a maximum possible score of 5, while the Side Effects and Medication Administration videos each had a maximum possible score of 4. We also evaluated parents' comprehension, confidence, usefulness, and satisfaction using a Likert scale ranging from (1) Strongly Agree to (5) Strongly Disagree for each of the three medication videos. Comprehension was assessed via knowledge scores, confidence via comfort in applying knowledge (e.g., medication administration), usefulness via learning new facts or skills, and satisfaction via perceptions of content quality and likelihood of recommending the videos to other parents. Lower scores indicated greater satisfaction with video content.

### 2.4 Analysis

To statistically compare sociodemographic characteristics between English- and Spanish-speaking cohorts, we used Chi-square tests or Fisher's exact tests for categorical data, and Mann-Whitney *U* tests for continuous data. Wilcoxon signed-rank test was used to compare pre-video and post-video median knowledge scores. McNemar's test was used for individual questions to identify any statistically significant differences in the proportion of participants who answered each question correctly pre-video compared to post-video. Binomial regression was performed on participants' knowledge scores, comparing the change in scores between English-language video viewers and Spanish-language video viewers controlling for the baseline scores in each question domain. *P*-value of less than 0.05 was considered statistically significant.

## 3 Results

### 3.1 Demographics

Nineteen parents were recruited for the focus groups. Ten parents reported English, and nine parents reported Spanish as their primary language. Most parents were women (79%) with the median age of 34 years. Parents' highest level of education varied, with 5% having less than a high school diploma, 36.8% completing high school, 36.8% having some college or an associate degree, and 21% holding a bachelor's degree or higher.

One hundred participants watched the three educational videos and completed both the pre-video and post-video surveys. Participant characteristics are summarized in Table 2. Eighty-seven percent of the participants were female primary caretakers compared with 13% who were male. The median age of participants was 35 years with a standard deviation of 8.05 years. Race and ethnicity were combined into a single variable and participants could select multiple categories or provide a free-text response to better reflect self-identification. Participants who identified as Latino or Hispanic comprised 63%, followed by those identifying as Caucasian at 19%, and those identifying as African American at 11%. Participants reported their highest degree or level of education completed as high school (33%), some college (33%), or bachelor's degree (24%). Compared to the English-language video viewers, Spanish-language video viewers were more likely to report high school as their highest degree or level of education completed.

**Table 2:** Sociodemographic Characteristics of English- and Spanish-Speaking Parent Participants.

Number of participants (n)	All participants (*n* = 100)	English-speaking participants (*n* = 50)	Spanish-speaking participants (n = 50)	*p*-value⁎⁎
Language, N (%)⁎				
English	50 (50.00%)	50 (100.00%)	0 (0.00%)	
Spanish	50 (50.00%)	0 (0.00%)	50 (100.00%)	
Gender, N (%)				
Male	13 (13.00%)	11 (22.00%)	2 (4.00%)	**0.015**
Female	87 (87.00%)	39 (78.00%)	48 (96.00%)	
Years of age, Median (SD)+				
	35 (8.05)	35.50 (6.81)	33 (9.10)	0.332
Self-reported ethnic background, N (%)				
Caucasian	19 (19.00%)	18 (36.00%)	1 (2.00%)	**< 0.001**
African American	11 (11.00%)	11 (22.00%)	0 (0.00%)	
Latino or Hispanic	63 (63.00%)	14 (28.00%)	49 (98.00%)	
Asian or Pacific Islander	5 (5.00%)	5 (10.00%)	0 (0.00%)	
Other or prefer not to say	2 (2.00%)	2 (4.00%)	0 (0.00%)	
Highest degree or level of education completed, N (%)				
High school	33 (33.00%)	9 (18.00%)	24 (48.00%)	**0.019**
Some college	33 (33.00%)	21 (42.00%	12 (24.00%)	
Bachelor's degree	24 (24.00%)	14 (28.00%)	10 (20.00%)	
Master's degree or higher	6 (6.00%)	3 (6.00%)	3 (6.00%)	
Prefer not to say	4 (4.00%)	3 (6.00%)	1 (2.00%)	

⁎ Data are presented as counts of participants and percentages, N (%), for categorical variables.

+ Data are presented as medians and standard deviations, Median (SD), for continuous variables. ⁎⁎ Bolded values denote statistical significance (*p* < 0.05). Statistical comparisons between English- and Spanish-speaking cohorts were performed using Chi-square tests or Fisher's exact tests for categorical data, and Mann-Whitney *U* tests for continuous data.

### 3.2 Change in knowledge

Parents' knowledge scores increased significantly after viewing all three educational videos (Table 3). Among both English-language and Spanish-language video viewers, median post-video knowledge scores were significantly higher than pre-video scores across all three educational videos (*p* < 0.001). When all participants were analyzed together, median scores increased across each knowledge domain: Medication Instructions: from 2.00 (2.00–3.00) to 4.00 (3.00–5.00); Side Effects, from 2.00 (1.75–3.00) to 3.00 (3.00–4.00); and Medication Administration, from 2.00 (1.00–3.00) to 3.00 (2.00–4.00).

**Table 3:** Comparison of Pre-Video and Post-Video Pediatric Medication Knowledge Scores Based on Parent Participant Language.

		Pre-video knowledge score⁎	Post-video knowledge score⁎	P-value
English-speaking participants (n)		(*n* = 50)	(n = 50)	
	Medication Instruction			
	Median (Q1-Q3)	3.00 (2.00–3.00)	5.00 (4.00–5.00)	< 0.001
	Medication Side Effects			
	Median (Q1-Q3)	2.00 (1.25–3.00)	3.50 (3.00–4.00)	< 0.001
	Medication Administration			
	Median (Q1-Q3)	2.00 (1.00–3.00)	4.00 (2.00–4.00)	< 0.001
Spanish-speaking participants (n)		(n = 50)	(n = 50)	
	Medication Instruction			
	Median (Q1-Q3)	2.00 (2.00–3.00)	4.00 (3.00–5.00)	< 0.001
	Medication Side Effects			
	Median (Q1-Q3)	2.00 (2.00–3.00)	3.00 (3.00–4.00)	< 0.001
	Medication Administration			
	Median (Q1-Q3)	2.00 (1.00–3.00)	3.00 (2.00–4.00)	< 0.001

*P*-values evaluate the change in knowledge scores between pre-video (prior to parent participants having watched the medication videos) and post-video (after parent participants have watched the medication videos). Wilcoxon signed-rank test for paired non-parametric data was used.

⁎ Data represent median knowledge test scores (maximum score of 5, excepts for Medication Side Effects video which has a maximum score of 4) and interquartile range (IQR) stretching from the 25th percentile (Q1) to the 75th percentile (Q3).

To evaluate the performance of individual knowledge questions, we conducted an item-level analysis. No single question was answered incorrectly by all participants, suggesting that no individual question was systematically misunderstood.

Knowledge gains were similar for English-language and Spanish-language video viewers. Binomial regression analyses controlling for baseline knowledge scores showed no significant differences in knowledge improvement between language groups across any of the three educational videos (Table 4).

**Table 4:** Binomial Logistic Regression Analysis Evaluating the Effect of Language Group on Post-Video Knowledge Score Improvement Controlling for Baseline Scores.

Variable	exp(β)⁎	95% C.I.+	*p*-value
Medication Instruction			
Spanish speaker	0.48	(0.164, 1.33)	0.168
Pre-test score	2.31	(0.243, 25.31)	0.475
Medication Side Effects			
Spanish speaker	0.91	(0.394, 2.087)	0.820
Pre-test score	2.88	(0.453, 19.62)	0.267
Medication Administration			
Spanish speaker	0.78	(0.344, 1.759)	0.547
Pre-test score	5.81	(0.958, 39.003)	0.061

Statistical modeling was performed using binomial regression to estimate the likelihood of knowledge score improvement between language groups while controlling for baseline pre-test performance. Significance is defined at *p* < 0.05.

⁎ exp.(β) denotes the exponentiated beta coefficient (Odds Ratio).

+ Values represent the 95% Confidence Interval (C.I.) for the estimate.

### 3.3 Parents' comprehension, confidence, and perception of educational video utility

After watching the three educational videos, participants provided feedback on the video quality in the form of a Likert scale, with options ranging from “strongly agree” (1) to “strongly disagree” (5). Table 5 shows that all three videos received strongly favorable feedback on comprehension, confidence, usefulness, and satisfaction. For the medication administration video, Spanish-language video viewers were more likely to report satisfaction by recommending the educational video to other parents compared to English-language video viewers.

**Table 5:** Parent Participant Perceptions of Video Quality, Comfort, and Usefulness Based on Parent Participant Language.

Number of participants (n)	English-speaking participants (*n* = 50)	Spanish-speaking participants (n = 50)	p-value
The information in the video was easy to understand, Median Likert value (Q1-Q3)⁎			
Medication Instruction	1.00 (1.00–2.00)	1.00 (1.00–2.00)	0.810
Medication Side Effects	1.00 (1.00–2.00)	1.00 (1.00–1.00)	0.095
Medication Administration	1.00 (1.00–2.00)	1.00 (1.00–2.00)	0.676
I feel more comfortable giving my child medications after watching this video, Median Likert value (Q1-Q3)			
Medication Instruction	1.00 (1.00–2.00)	1.00 (1.00–2.00)	0.432
Medication Side Effects	1.00 (1.00–2.00)	1.00 (1.00–2.00)	0.699
Medication Administration	1.00 (1.00–2.00)	1.00 (1.00–2.00)	0.488
I learned a new useful fact or skill after watching this video, Median Likert value (Q1-Q3)			
Medication Instruction	1.00 (1.00–2.00)	1.00 (1.00–2.00)	0.725
Medication Side Effects	1.00 (1.00–2.00)	1.00 (1.00–1.75)	0.161
Medication Administration	1.00 (1.00–2.00)	1.00 (1.00–1.00)	0.371
I would recommend this video to other parents, Median Likert value (Q1-Q3)			
Medication Instruction	1.00 (1.00–2.00)	1.00 (1.00–1.00)	0.363
Medication Side Effects	1.00 (1.00–2.00)	1.00 (1.00–1.00)	0.233
Medication Administration	1.00 (1.00–2.00)	1.00 (1.00–1.00)	0.013

⁎ Values are based on a 5-point Likert scale, where 1 = “Strongly Agree”, 2 = “Agree”, 3 = “Neutral”, 4 = “Disagree”, and 5 = “Strongly Disagree”. Lower median score values indicate higher parental satisfaction and agreement with the statement. Data are presented as Median Likert values and interquartile range (IQR) spanning the 25th percentile (Q1) to the 75th percentile (Q3). Between-group comparisons (English-speaking vs. Spanish-speaking parent participants) were evaluated using the non-parametric Mann-Whitney *U* test (or Wilcoxon rank-sum test). Significance is defined at *p* < 0.05.

## 4 Discussion and conclusion

### 4.1 Discussion

This study aimed to address the significant health disparities associated with lower health literacy rates among parents in underserved communities by developing and evaluating video-based educational resources focused on pediatric medication management. With 25% to 43% of children in the United States having been reported to take medications to treat various health issues, the content of our videos are applicable to millions of children. [48] Our study results demonstrate the effectiveness of these video-based resources not only in improving parents' comprehension and knowledge of key medication-related concepts but also in instilling confidence in applying medications, all of which are critical steps toward reducing adverse health outcomes in children. These findings are particularly encouraging given the diverse linguistic and cultural backgrounds of families in our communities. The consistency of the positive feedback across both English-language and Spanish-language video viewers suggests that the videos were culturally relevant and accessible, which is crucial for reaching a broader audience in underserved areas. By increasing access to concise, comprehensible medication information, gaps in health literacy could decrease, subsequently reducing duration of illness, and enhancing health management.

To improve parents' accessibility to medication education, we chose video-based education given its multiple advantages. First, videos enable parents to receive education at their convenience, allowing them to engage with the information when they have the time and energy to focus. This approach contrasts with verbal forms of teaching that are planned according to healthcare provider schedules. Second, video-based education allows for repeat viewing by multiple caregivers who could be involved in the child's care, enabling consistent messages to be delivered. Unlike written materials, videos can convey complex information through visual and auditory means, making it easier for parents with lower health literacy or lower English proficiency to understand and retain key concepts. The dynamic nature of videos allows for the demonstration of procedures, such as medication administration techniques, which can be more effectively learned through observation rather than reading instructions. Additionally, videos can incorporate engaging elements like animations, voiceovers, and real-life scenarios that help maintain the viewer's attention and improve comprehension. For individuals with low health literacy, videos provide an accessible alternative that can be paused and rewatched, enhancing learning flexibility.

A key factor in the success of these educational videos was the use of a patient-centric product development approach. By involving parents directly in the development process through iterative focus groups and feedback sessions, we were able to tailor the content to meet the specific needs and preferences of the target audience. Culturally-tailored education has been shown in literature to be the preferred form of education by patients and effective at improving patient self-management. [18], [49], [50], [51], [52] In our study, we were able to demonstrate improvement in post-video knowledge scores and there was no statistical difference in score improvement between the English-language and Spanish-language video viewers. This suggests that regardless of the video language version, the videos successfully conveyed the same quality and depth of information to parents of both groups. The active participation of parents in the creation and refinement of the videos thus played a crucial role in producing high-quality resources that resonate with the community.

Despite the positive outcomes of this study, several limitations should be considered. First, the sample size of our iterative development process, though diverse, was relatively small. Second, we conducted focus group recruitment in a few underserved neighborhoods with unique patient sociodemographic factors, which may not be representative of the entire Spanish-speaking community. However, these views are often underrepresented and require exploration to improve the health outcomes of these families. Additionally, while the videos were available in both English and Spanish, the study did not explore the effectiveness of these educational resources among non-Spanish-speaking immigrant communities or parents with different cultural backgrounds, potentially limiting their broader applicability. Our study also did not perform a formal health literacy assessment on our parent participants; however, the low median pre-test knowledge scores across all three medication topics suggested that participants did not have a good understanding of medication instruction, side effects, or administration. Additionally, we did not exclude parents who have had experience managing the specific medications covered in the videos. It is possible that parents who had previously used these medications performed better on the post-video knowledge assessment, potentially influencing the measured learning outcomes. Given that the medications addressed in the videos are widely encountered in routine pediatric care, excluding parents with prior exposure would have substantially limited the eligible sample and potentially resulted in a self-selected population unrepresentative of typical families. Because the educational videos were designed for general use among parents regardless of prior medication experience, we aimed to recruit a sample reflective of the broader population the materials are intended to serve. Lastly, our study primarily relied on self-reported measures of comprehension, confidence, usefulness, and satisfaction, which may be subject to self-reported bias. There is also the potential influence of participant incentives on video viewing, as paid participation may not reflect voluntary uptake in real-world settings. The study also lacked assessment of long-term knowledge retention, which is critical for sustained impact, and did not directly measure behavior change, such as actual improvements in medication administration practices. Thus, this is an area that should be addressed in future studies.

### 4.2 Innovation

The innovation of this study lies in both our methodology and educational material. PCPD has been increasingly recognized as an important framework within pharmaceutical and medical device development for incorporating patient perspectives into product design and optimizing usability, acceptability, and patient outcomes. [33] Particularly in pediatric populations, studies have highlighted the importance of pediatric-centricity in medicinal product development, including clear instructions for caregivers to facilitate adherence and avoid medication errors. [53], [54], [55] Despite this growing emphasis, existing studies that coproduced educational materials with pediatric patients have primarily focused on diseases and genetic conditions rather than medication-related topics. [19], [20], [21] By operationalizing PCPD in the development of pediatric medication education videos, we systematically incorporated parents' feedback to ensure that the videos were not only effective educational tools but also culturally relevant, engaging, and easy to understand. Ultimately, our study introduces a replicable, theory-informed framework for translating consumer input into practical, end-user materials.

In addition to this methodological innovation, our study also contributes novel educational content. Unlike other medication videos, our videos address foundational pediatric medication topics, including frequency of administration, side effects, and using effective strategies to administer medications, which parents identified as key knowledge gaps in our previous study. [56] By shifting from a provider-driven, top-down model to a co-creation approach, we were able to include educational content that has not been previously published. For example, to our knowledge, most pediatric medication administration studies have been focused on the use of standardized dosing tools. [28], [29], [30], [57], [58] Our video, however, addresses practical medication administration strategies, including using appropriate language when administering medication, offering children choices to optimize adherence, and helping children learn to swallow pills at home. By identifying and prioritizing information gaps that have not been addressed in existing literature, our work introduces both novel educational content and a transferable, replicable methodological approach for future community health education initiatives.

Together, these contributions advance the field of patient and family education by demonstrating how PCPD can be adapted as a rigorous framework for medication education development while simultaneously producing novel educational resources that address unmet parent information needs. The approach offers a transferable model for future patient-centered educational interventions and provides practical guidance for researchers seeking to incorporate stakeholder perspectives into the development of health education materials.

### 4.3 Conclusion

This study successfully developed and validated video-based educational resources that are both comprehensible and valuable to parents in underserved communities. By continuing to refine and expand these resources, we can make meaningful progress in addressing health literacy gaps and improving pediatric health outcomes. Looking forward, the next steps will involve expanding the availability of these educational videos to include additional languages and tailoring them to address specific cultural nuances. This includes monitoring real-world uptakes through metrics like click counts and viewing time on hosted platforms, assessing longitudinal knowledge retention through follow-up surveys with this cohort, and evaluating behavior change via observational studies or self-reported adherence measures. This expansion is essential to further reduce health disparities and improve health outcomes in diverse populations.

## CRediT authorship contribution statement

**Tiranun Rungvivatjarus:** Writing – review & editing, Writing – original draft, Investigation, Funding acquisition, Formal analysis, Data curation, Conceptualization. **Nayely Cazares:** Writing – review & editing, Writing – original draft, Visualization, Project administration, Investigation, Formal analysis. **Letzy Vargas:** Writing – review & editing, Writing – original draft, Visualization, Project administration, Investigation, Formal analysis. **Elizabeth Haddad:** Writing – review & editing, Investigation. **Michael Henne:** Writing – review & editing, Writing – original draft, Project administration, Investigation, Formal analysis. **Gale Romanowski:** Writing – review & editing, Investigation, Data curation. **Kyung E. Rhee:** Writing – review & editing, Supervision, Methodology, Conceptualization.

## Funding sources

The work was supported by the 10.13039/100005295American Academy of Pediatrics (AAP) Community Access to Child Health (CATCH) Grant. The AAP did not have any involvement in the study design, collection, data analysis, interpretation of data, writing of the report or decision to submit the article for publication.

## Declaration of competing interest

The authors declare the following financial interests/personal relationships which may be considered as potential competing interests:

**Table:** 

Tiranun Rungvivatjarus reports financial support was provided by American Academy of Pediatrics. If there are other authors, they declare that they have no known competing financial interests or personal relationships that could have appeared to influence the work reported in this paper.
