# Supplementary material for: Developing Pediatric medication education for underserved families: A patient-centric product development approach focusing on medication instructions, side effects, and administration techniques
Source: PEC Innov. 2026 Sep 28;9:100507. doi: 10.1016/j.pecinn.2026.100507 (PMC13654438; doi:10.1016/j.pecinn.2026.100507)
Supplement: Supplementary file 1 [file mmc1.docx]

Supplemental Material

**Medication Knowledge Questions**

Medication Instruction

1. For medications taken twice daily, what is the minimum wait before giving your child the second dose?

- 4 hours
- 6 hours
- 8 hours
- 12 hours

2. If your child is prescribed a medication that is three times a day, what is the most ideal medication schedule?

- The first dose after lunch, the second dose during dinner, and the last dose at bedtime
- The first dose before breakfast, the second dose during lunch, and the last dose before bed
- The first dose before breakfast, the second dose during lunch, and the last dose 3 hours after lunch
- None of the above

3. If your child is prescribed a medication four times a	day, at least how many hours should you wait between?

- 2 hours
- 3 hours
- 4 hours
- 6 hours

4. If a medication is recommended to be taken on an empty stomach, what is the ideal spacing around meals:

- 1 hour after a meal
- 1 hour before a meal
- 15 minutes after a meal
- 15 minutes before meal

5. Which of the following is true about as needed medications (example: tylenol, motrin)?

- Since these medications are as needed, you can give the medication as often as you need per day
- As needed medications have no side effects
- If the child is not experiencing the symptoms the medication treats, you should still give them the medication
- Take note of the timing between doses and the maximum dose they can take for each as needed medication

Medication Administration

1. Which of the following are food(s) that you can mix	medication with to make it taste better for your child?

- Pudding
- Apple sauce
- Yogurt
- Popsicles
- All of the above

2. All of the following are effective recommended methods for giving your child medication, EXCEPT:

- Giving your child choices of how and when they take their medication
- Using comfort holds
- Hide medication in your child's food without telling him/her
- Explaining to your child that the medication will help him/her feel better

3. Which of the following phrases is not an effective way to talk to your child about their medication?

- "Take this medication and you'll feel better"
- "You are awesome for taking your medication"
- "If you do not take this medication, you cannot go to the park today"
- "Take this medication and then we can go play"

4. Which of the following is a way(s) your pharmacist can help you with your child's medication?

- The pharmacist can let you know if the medication can be given without the capsule itself
- The pharmacist can help you with flavoring options
- The pharmacist can provide ideas to make the medication taste better
- All of the above

Medication Side Effects

1. All of the following side effects of amoxicillin and cephalexin require immediate MEDICAL ATTENTION EXCEPTS:

- Severe diarrhea
- Bloody diarrhea
- Stomach pain/cramps
- Some diarrhea

2. Which of the following is true about acetaminophen?

- Do not exceed more than 3 doses a day.
- Acetaminophen in combination cold products does NOT count towards your child's daily acetaminophen total dosage.
- Acetaminophen is safe to store in sight of children
- Acetaminophen overdose can result in liver toxicity

3. Which of the following will improve the effect of Polyethylene Glycol, i.e. Miralax?

- Take with a full meal
- Take on an empty stomach
- Take without water
  - Take with a full glass of water

4. Which medication can result in anxiety, fast heartbeat, and shakiness?

- Cephalexin
- Ondansetron
- Cetirizine/Loratadine
- Albuterol
